# High neutrophil‐to‐lymphocyte ratio is a predictor of poor short‐term outcome in patients with mild acute ischemic stroke receiving intravenous thrombolysis

**DOI:** 10.1002/brb3.1857

**Published:** 2020-09-27

**Authors:** Yong‐Lin Liu, Zhi‐Qiang Wu, Jian‐Feng Qu, Dong‐Hai Qiu, Gen‐Pei Luo, Han‐Peng Yin, Xue‐Wen Fang, Fang Wang, Yang‐Kun Chen

**Affiliations:** ^1^ Department of Neurology Dongguan People’s Hospital (Affiliated Dongguan Hospital South Medical University) Dongguan China; ^2^ Department of Radiology Dongguan People’s Hospital Dongguan China

**Keywords:** acute ischemic stroke, intravenous thrombolysis, neutrophil‐to‐lymphocyte ratio, outcome

## Abstract

**Introduction:**

Very few studies have investigated the specific relationship between neutrophil‐to‐lymphocyte ratio (NLR) and the short‐term outcomes of patients suffering from mild acute ischemic stroke (AIS) and receiving intravenous thrombolysis (IVT). This study aimed to investigate whether a high NLR is associated with a poor short‐term outcome in patients with mild AIS after IVT.

**Methods:**

We retrospectively analyzed data that were prospectively acquired from patients with AIS treated with IVT. Mild AIS was defined as a National Institutes of Health Stroke Scale (NIHSS) score ≤ 7 on admission. The NLR was based on a blood test performed prior to IVT and was classified as ‘high’ when exceeding the 75th percentile. Follow‐ups were performed at discharge and 3 months after onset. A poor outcome was defined as a modified Rankin scale (mRS) ≥3.

**Results:**

A total of 192 patients were included in this study. The median NLR was 3.0 (interquartile range [IQR]: 2.0–3.9). Fifty‐one patients (26.6%) had a high NLR (≥3.9) on admission. Forty‐one patients (21.4%) had a poor outcome at discharge, while 34 patients (17.7%) had a poor outcome at 3 months. Patients with a poor outcome at discharge, and at 3 months after onset, were more likely to have a high NLR at discharge (42.9% vs. 21.9%; *p* = .005) and at 3 months (44.1% vs. 22.8%; *p* = .011), compared with those with a better outcome. After adjustment for NIHSS score on admission, ipsilateral severe intracranial large artery occlusion, and atrial fibrillation, logistic regression analyses revealed that a high NLR was a significant predictor of poor outcome at discharge and at 3 months after onset.

**Conclusions:**

A high NLR on admission could be a useful marker for predicting poor short‐term outcome in patients with mild AIS following IVT.

## Graphical abstract

To the best of our knowledge, few studies have focused on the specific relationship between neutrophil‐to‐lymphocyte ratio (NLR) and the short‐term prognosis of patients with mild acute ischemic stroke (AIS) and treated with intravenous thrombolysis (IVT). Our study evaluate whether an increased NLR was predictive of a poor short‐term outcome in patients suffering from mild AIS and treated with IVT. A high NLR (≥3.9) was associated with a poor outcome in patients with mild AIS treated with IVT, at both discharge and 3 months after onset.

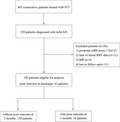

## 1 INTRODUCTION

Intravenous thrombolysis (IVT) with recombinant tissue plasminogen activator (r‐tPA) is an effective treatment for patients with acute ischemic stroke (AIS) if administered within 4.5 hr of stroke onset (National Institute of Neurological Disorders and Stroke rt‐PA Stroke Study Group, 1995;Hacke et al., 2008; NINDS rt‐PA Stroke Study Group, 1995). However, according to previous studies, only approximately 50% of patients (45.0%–52.4%) receiving IVT experience a favorable outcome (Ahmed et al., 2010; Hacke et al., 2008; Lees et al., 2010). A previous study reported that 54.6% of AIS patients presented as mild AIS (Dhamoon et al., 2009). Other reports show that 30% of patients with mild AIS experience a poor outcome following IVT (Emberson et al., 2014; Romano et al., 2015; Yu et al., 2015). Therefore, it is very important to identify factors associated with poor outcome in patients with mild AIS following IVT. A range of such factors have been suggested in previous studies, including age, diabetic status, National Institutes of Health Stroke Scale (NIHSS) score on admission (Romano et al., 2015), and large vessel occlusion (Nedeltchev et al., 2007). In mild AIS, the weighting of neurological deficits for poor outcome might be reduced by a lower NIHSS score. Therefore, potential risk factors other than NIHSS score, such as serum biomarkers, may play a more important role in the outcome of mild AIS. However, only a very limited number of previous studies have focused on serum biomarkers. Poststroke inflammatory response (IR) has been shown to play an important role in secondary brain injury (Jenny et al., 2019; Shi et al., 2019; Urday et al., 2015). Previous research has also shown that IR is related to a poor outcome in patients suffering from AIS (Shi et al., 2019). Peripheral circulating leukocytes, recruited by immune mediators, are known to be critical in the processes that cause neuronal damage following stroke (Kim et al., 2016; Rayasam et al., 2018). The neutrophil‐to‐lymphocyte ratio (NLR), which reflects the number of neutrophils and lymphocytes in the peripheral circulation, has previously been shown to be related to a poor functional outcome in patients suffering from acute intracerebral hemorrhage (Lattanzi et al., 2016), subarachnoid hemorrhage (Tao et al., 2017), and AIS (Giede‐Jeppe et al., 2019; Qun et al., 2017; Wang et al., 2019). However, previous research investigating the relationship between NLR and poor outcome in patients suffering from AIS did not exclude patients with in‐hospital pneumonia (IHP), a potential confounder. In addition, to the best of our knowledge, few studies have focused on the specific relationship between NLR and the short‐term prognosis of patients with mild AIS and treated with IVT. In the present study, we aimed to evaluate whether an increased NLR was predictive of a poor short‐term outcome in patients suffering from mild AIS and treated with IVT.

## 2 MATERIALS AND METHODS

### 2.1 Patients

We prospectively recruited AIS patients receiving r‐tPA IVT therapy in Dongguan People's Hospital between January 1, 2016, and October 31, 2019. We then retrospectively analyzed data from all subjects receiving IVT. Mild AIS was defined as an NIHSS score on admission ≤ 7 (Kirchhof et al., 2017). The inclusion criteria were as follows: (a) age ≥ 18 years; (b) AIS confirmed by magnetic resonance imaging (MRI); (c) onset of stroke symptoms within 4.5 hr and treated with r‐tPA; and (d) NIHSS score on admission ≤ 7. Patients were excluded in accordance with the following criteria: (a) missing data from routine blood tests (RBTs) on admission; (b) a prestroke modified Rankin Scale (mRS) score ≥ 2; (c) a history of infection within the 2 weeks prior to stroke, or the occurrence of IHP, or other infections, within 1 week of onset; (d) a history of long‐term immunosuppressant drug use; (e) hematological or rheumatic disorders; (f) additional endovascular therapy following IVT; and (g) loss to follow‐up. This study was approved by the hospital ethics committee (approval number: KYKT2018‐002). Each subject provided informed consent in accordance with the Declaration of Helsinki.

### 2.2 Data collection

NIHSS score and onset to treatment time (OTT) were acquired for each patient, as well as a range of demographic data (age, gender), history of hypertension, diabetic status, smoking status, atrial fibrillation (AF), and previous stroke history. Initial counts for white blood cells, neutrophils, lymphocytes, and platelets prior to IVT were also acquired; these data were used to calculate the NLR. We found that the NLR data was not normally distributed (Kolmogorov–Smirnov test; *p *＜ .001). Consequently, we classified NLR data into high and low groups. The NLR was defined as ‘high’ if it exceeded the 75th percentile (≥3.9 in our cohort of subjects).

### 2.3 Clinical outcome

Functional status was assessed by mRS score at discharge and at 3 months after the onset of stroke. All patients were followed up; their mRS score was reassessed at 3 months via a face‐to‐face interview, or via telephone. A poor outcome was defined as an mRS score ≥ 3.

### 2.4 Imaging analysis

Brain magnetic resonance imaging (MRI) was performed for each patient, including T1‐weighted imaging (T1WI), T2‐weighted imaging (T2WI), fluid‐attenuated inversion recovery (FLAIR), diffusion‐weighted imaging (DWI), and three‐dimensional time‐of‐flight MRA (3D‐TOF‐MRA). All scanning was carried out with a 3.0 T system (Skyra, Siemens Medical, Erlangen, Germany) within 72 hr of admission. The MRI parameters used for these scans were reported in our previous study (Li et al., 2019).

Ipsilateral severe intracranial large artery stenosis (ISILAS) and ipsilateral severe intracranial large artery occlusion (ISILAO) were evaluated by 3D‐TOF‐MRA. ISILAS was defined as cases involving the internal carotid artery, or the M1 segment of the middle cerebral artery, ipsilateral to the infarction with a diameter loss >70%; ISILAO was defined as a loss of signal for distal blood flow ipsilateral to the infarction. The assessment was based on the Warfarin Aspirin Symptomatic Intracranial Disease criteria (WASID Study Group, 1998). Hemorrhagic transformation (HT) was defined as a secondary hemorrhage, within or away from the infarction area, which was evident as hyperdense lesions on repeated computed tomography (CT) scans 1 week after IVT.

Two experienced MRI‐specialized neuroradiologists, who were blinded to clinical information, independently evaluated the imaging findings for each subject, including the severity of intracranial artery stenosis and HT.

### 2.5 Statistical analysis

Statistical analyses were conducted using SPSS for Windows (version 20.0, IBM Corp., Armonk, NY, USA). Continuous variables that were normally distributed are reported as means (with standard deviation), while non‐normally distributed variables are reported as a median and interquartile range (IQR). All subjects were divided into two groups with regards to a poor outcome at discharge or at 3 months. Variables were compared between the groups using *t* tests, Mann–Whitney *U* tests, Pearson *χ* ^2^‐tests, or Fisher's exact tests, as appropriate. Variables for which *p* < .05 in the univariate analysis were subsequently included in further binary multivariate logistic regressions. Statistical significance was defined as *p* < .05 (two‐sided).

## 3 RESULTS

### 3.1 Clinical characteristics of patients

**FIGURE 1:**
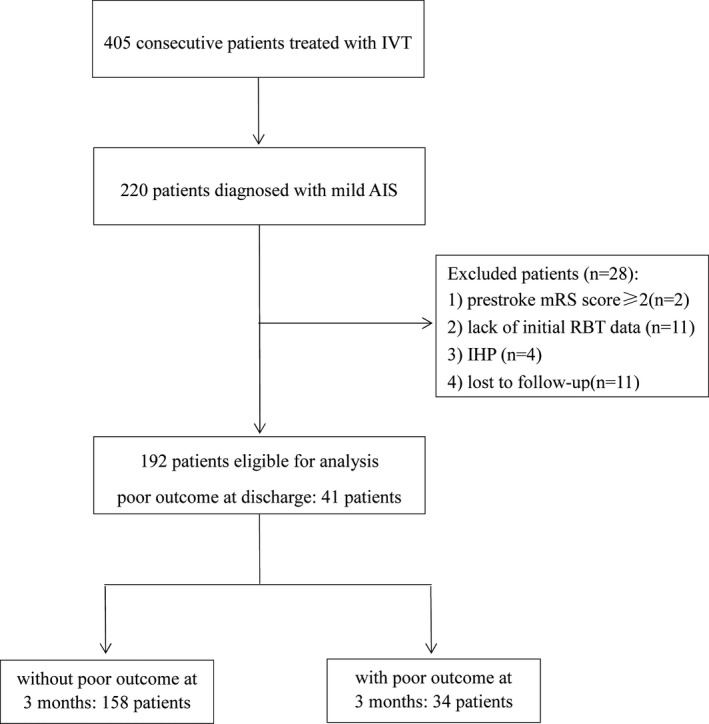
Diagram of the study recruitment Note: AIS, acute ischemic stroke; IHP, in‐hospital pneumonia; IVT, intravenous thrombolysis; mRS, modified Rankin scale; RBT, routine blood test

During the study period, 405 consecutive patients with AIS were treated with r‐tPA IVT within 4.5 hr of the onset of stroke. Of these 405 patients, 220 patients were diagnosed with mild AIS. Of these 220 patients, 28 patients were excluded for the following reasons: prestroke mRS score ≥ 2 (*n* = 2), lack of initial RBT data (*n* = 11), and IHP (*n* = 4); 11 patients were lost to follow‐up. Therefore, a total of 192 patients were included in our final analysis. A diagram depicting study recruitment is given in Figure 1.

The mean age of the 192 patients recruited into our final analysis was 60.8 ± 11.7 years; 138 (71.9%) of these were male. The median duration of hospitalization was 11.0 days (interquartile range [IQR]: 8.0–15.0 days). The mean OTT was 206.4 ± 57.1 min, and the median NIHSS score on admission was 5.0 (IQR: 3.0–6.8). The median NLR was 3.0 (IQR: 2.0–3.9). Fifty‐one patients (26.6%) had a high NLR on admission. All of the patients were alive at follow‐up. The demographic and clinical characteristics of the study cohort are shown in Table 1.

**TABLE 1:** Demographic and clinical characteristics of the study cohort

Characteristics	Mean(*SD*)/Median(IQR)/*n* (%) *n* = 192
Age (yr)	60.8 ± 11.7
Men (*n*, %)	138 (71.9%)
Hypertension (*n*, %)	148 (77.1%)
Diabetes mellitus (*n*, %)	52 (27.1%)
Smokers/ex‐smokers (*n*, %)	75 (39.1%)
Atrial fibrillation (*n*, %)	35 (18.2%)
Previous stroke (*n*, %)	29 (15.1%)
OTT (min)	206.4 ± 57.1
NIHSS score on admission	5.0 (3.0–6.8)^a^
Hospitalization duration (days)	11.0 (8.0–15.0)^a^
Poor outcome at discharge (*n*, %)	41 (21.4%)
Poor outcome at 3 months (*n*, %)	34 (17.7%)
Platelet counts (10^9^/L)	213.4 ± 58.5
WBC counts (10^9^/L)	8.1 ± 2.7
Neutrophil counts (10^9^/L)	5.4 ± 2.3
Lymphocyte counts (10^9^/L)	2.0 ± 0.9
NLR	3.0 (2.0–3.9)^a^
High NLR (*n*, %)	51 (26.6%)
BG on admission (mmol/L)	7.5 ± 3.1
ISILAS (*n*, %)	33 (17.2%)
ISILAO (*n*, %)	16 (8.3%)
Hemorrhagic transformation (*n*, %)	30 (15.6%)

Abbreviations: BG, blood glucose; ISILAO, ipsilateral severe intracranial large artery occlusion; ISILAS, ipsilateral severe intracranial large artery stenosis; NIHSS, National Institutes of Health Stroke Scale; NLR, neutrophil‐to‐lymphocyte ratio; OTT, onset to treatment time; WBC, white blood cell.

^a^ Median (25Q‐75Q).

### 3.2 Univariate analysis

Univariate analysis indicated that patients with a poor outcome at discharge had a higher NIHSS score and higher neutrophil counts on admission, as well as a greater prevalence of AF and ISILAO (*p* < .05). Patients with a poor outcome at 3 months had a higher NIHSS score on admission than those without a poor outcome, and a greater prevalence of AF and ISILAO (*p* < .05). Patients with a poor outcome at discharge and at 3 months were more likely to have a high NLR (discharge: 42.9% vs. 21.9%, *p* = .005; 3 months: 44.1% vs. 22.8%, *p* = .011), compared with those with a better outcome. The results of our univariate analysis are shown in Table 2.

**TABLE 2:** Risk factors for poor outcome on discharge and at 3 months after the onset of stroke, as determined by univariable analysis

Variable	Poor outcome at discharge		*p* value	Poor outcome at 3 months		*p* value
	With (*n* = 41)	Without (*n* = 151)		With (*n* = 34)	Without (*n* = 158)	
Age^a^ (yr)	61.2 (9.5)	60.7 (12.3)	.793	62.5 (9.6)	60.4 (12.1)	.335
Men^b^ (*n*, %)	30 (73.2%)	108 (71.5%)	.835	22 (64.7%)	116 (73.4%)	.305
Hypertension^b^ (*n*, %)	32 (78.0%)	116 (76.8%)	.868	25 (73.5%)	123 (77.8%)	.587
Diabetes^b^ (*n*, %)	8 (19.5%)	44 (29.1%)	.219	8 (23.5%)	44 (27.8%)	.607
Smokers/ex‐smokers^b^ (*n*, %)	15 (36.6%)	60(39.7%)	.714	10(29.4%)	65 (41.1%)	.204
Atrial fibrillation^b^ (*n*, %)	12 (29.3%)	23 (15.2%)	.039	11 (32.4%)	24 (15.2%)	.019
Previous stroke^b^ (*n*, %)	9 (22.0%)	20 (13.2%)	.167	8 (23.5%)	21 (13.3%)	.130
OTT^a^ (min)	209.8 ± 53.5	204.4 ± 59.2	.584	208.9 ± 53.1	205.4 ± 58.5	.565
NIHSS score on admission^c^	6.0 (5.0–7.0)	4.0 (3.0–6.0)	＜.001	6.0 (5.0–7.0)	4.0 (3.0–6.0)	＜.001
Platelet counts^a^ (10^9^/L)	220.6 (51.9)	211.5 (60.1)	.397	217.1 (46.4)	212.6 (60.8)	.696
WBC counts^a^ (10^9^/L)	8.4 (2.2)	8.0 (2.8)	.429	8.4 (2.2)	8.0 (2.7)	.476
Neutrophil counts^a^ (10^9^/L)	6.0 (1.9)	5.2 (2.3)	.018	5.8 (2.1)	5.3 (2.3)	.226
Lymphocyte counts^a^ (10^9^/L)	1.8 (0.7)	2.0 (0.9)	.118	1.9 (0.8)	2.0 (0.9)	.628
High NLR^b^ (*n*, %)	18 (43.9%)	33 (21.9%)	.005	15 (44.1%)	36 (22.8%)	.011
BG on admission^a^ (mmol/L)	7.0 (2.4)	7.6 (3.3)	.218	7.2 (2.6)	7.6 (3.2)	.560
ISILAS^b^ (*n*, %)	10 (24.4%)	23 (15.2%)	.168	7 (20.6%)	26 (16.5%)	.562
ISILAO^b^ (*n*, %)	7 (17.1%)	9 (6.0%)	.022	7 (20.6%)	9 (5.7%)	.004
HT^b^ (*n*, %)	10 (24.4%)	20 (13.2%)	.081	8 (23.5%)	22 (13.9%)	.162

Note

Abbreviations: BG, blood glucose; HT, hemorrhagic transformation; ISILAO, ipsilateral severe intracranial large artery occlusion; ISILAS, ipsilateral severe intracranial large artery stenosis; NIHSS, National Institutes of Health Stroke Scale; NLR, neutrophil‐to‐lymphocyte ratio; OTT, onset to treatment time; WBC, white blood cell.

^a^ Mean(*SD*), *t* test.

^b^ n (%), chi‐square test.

^c^ Mann–Whitney *U* test.

### 3.3 Multivariate logistic regression analyses

Variables that showed significant differences between the two groups in the univariable analysis were subsequently entered into a logistic regression model. Because neutrophil counts and NLR were highly correlated (*r* = .797), two models were used for the logistic regression analysis for a poor outcome at discharge. In model 1 (featuring neutrophil counts), NIHSS score on admission (odds ratio [OR] = 1.681, 95% confidence interval [CI] = 1.316–2.148, *p < *.001), and ISILAO (OR = 3.435, 95% CI = 1.086–10.850, *p = *.036), were significantly associated with a poor outcome at discharge, while neutrophil count (*p* = .103) was not. In model 2 (featuring high NLR), NIHSS score on admission (OR = 1.657, 95% CI = 1.296–2.118, *p *＜ .001), ISILAO (OR = 3.605, 95% CI = 1.109–11.718, *p* = .033), and a high NLR (OR = 2.809, 95% CI = 1.271–6.208, *p* = .011), were identified as predictors for a poor outcome. In terms of outcome at 3 months after the onset of stroke, logistic regression demonstrated that NIHSS score on admission (OR = 1.664, 95% CI = 1.272–2.177, *p *＜ .001), ISILAO (OR = 4.946, 95% CI = 1.499–16.323, *p* = .009), and a high NLR (OR = 2.660, 95% CI = 1.146–6.177, *p* = .023), were significantly correlated with a poor outcome. The results of our multivariate logistic regression analyses are shown in Tables 3 and 4.

**TABLE 3:** The identification of predictors of poor outcome at discharge, by multivariate logistic regression

Variable	Poor outcome on discharge	
	*OR* (95% CI)	*p* value
Model 1 (featuring neutrophil counts)		
Atrial fibrillation	1.742 (0.720–4.213)	.218
NIHSS score on admission	1.681 (1.316–2.148)	＜.001
Neutrophil counts	1.141 (0.973–1.338)	.103
ISILAO	3.435 (1.086–10.850)	.036
Model 2 (featuring high NLR counts)		
Atrial fibrillation	1.551 (0.631–3.817)	.339
NIHSS score on admission	1.657 (1.296–2.118)	＜.001
High NLR	2.809 (1.271–6.208)	.011
ISILAO	3.605 (1.109–11.718)	.033

Note

Abbreviations: CI, confidence interval; ISILAO, ipsilateral severe intracranial large artery occlusion; NIHSS, National Institutes of Health Stroke Scale; NLR, Neutrophil‐to‐Lymphocyte Ratio; OR, odds ratio.

**TABLE 4:** The identification of predictors of poor outcome at 3 months after the onset of stroke by multivariate logistic regression

Variable	Poor outcome at 3 months	
	*OR* (95% CI)	*p* value
Atrial fibrillation	1.864 (0.728–4.775)	.194
NIHSS score on admission	1.664 (1.272–2.177)	＜.001
High NLR	2.660 (1.146–6.177)	.023
ISILAO	4.946 (1.499–16.323)	.009

Note

Abbreviations: CI, confidence interval; ISILAO, ipsilateral severe intracranial large artery occlusion; NIHSS, National Institutes of Health Stroke Scale; NLR, Neutrophil‐to‐Lymphocyte Ratio; OR, odds ratio.

## 4 DISCUSSION

Our analyses demonstrated that a high NLR (≥3.9) was associated with a poor outcome in patients with mild AIS treated with IVT, at both discharge and 3 months after onset, with an OR of 2.731 and 2.515, respectively. This observation has rarely been reported in previous studies.

IR following AIS has been proven to be related to secondary brain injury following the primary brain injury caused by the infarct (Jenny et al., 2019; Shi et al., 2019; Urday et al., 2015). According to previous studies, IR can exacerbate stroke and is associated with a poor outcome (De Meyer et al., 2016; Gan et al., 2014; Urday et al., 2015). IR can be triggered within minutes by a stroke event, and persist for days to weeks, or even longer (Beez et al., 2017; Urday et al., 2015). Disruption of the blood–brain barrier (BBB) takes place early after a stroke and facilitates the infiltration of peripheral leukocytes to the injured brain (Giraud et al., 2015). Leukocytes play an important role in IR (Kim et al., 2016; Rayasam et al., 2018) by releasing inflammatory cytokines at the site of injury, including tumor necrosis factor‐α, interleukin‐1 beta, and interleukin‐6 (Tuttolomondo et al., 2008). Neutrophils, a specialist form of leukocyte, have been shown to be involved in tissue damage in the brain following stroke (Aronowski & Roy‐O'Reilly, 2019; Schäbitz & Minnerup, 2019). There are several hypotheses relating to how neutrophils might exert negative effects in brain injury. For example, it is possible that neutrophils cause a physical blockade within the microvascular network, resulting in a further reduction in cerebral blood flow (2). Alternatively, neutrophils may directly enter the brain parenchyma, resulting in the release of granules containing antimicrobial enzymes and chemical species that can cause further aggravation to the injured brain tissue (Jickling et al., 2015; Kalimo et al., 2013). Another study reported that an increased concentration of neutrophils can result in the enhanced expression of matrix metalloproteinase‐9 (Justicia et al., 2003), a protein associated with BBB damage and brain injury (Jickling et al., 2014; Rosell et al., 2008; Zhang et al., 2003). Lymphocytes are also known to play important roles in inflammation (Frangogiannis et al., 2002; Schwartz & Moalem, 2001); however, the precise effects depend on the subtype of lymphocytes. Some lymphocytes are known to be neuroprotective (Liesz et al., 2013; Ren et al., 2011), while others have been shown to exacerbate inflammation (Kim et al., 2014; Liesz et al., 2015). NLR is regarded as a useful marker that can simultaneously reflect the negative effects of neutrophils and the positive effects of lymphocytes in stroke patients (Gibson et al., 2010; Gökhan et al., 2013). An increase in NLR has also been found to be a useful prognostic predictor in patients suffering from AIS (Giede‐Jeppe et al., 2019; Qun et al., 2017; Wang et al., 2019). In the present study, neither neutrophils nor lymphocytes had significant effects. However, because both neutrophil counts (*r* = .797) and lymphocytes (*r* = −.539) were highly correlated with NLR, our findings were in accordance with those of previous studies. NLR is easily evaluated by RBT, making it an economic and effective marker, even in regional hospitals.

NLR can be influenced by infections such as pneumonia and previous research has shown that IHP is related to a poor outcome in patients with AIS;^45^ therefore, IHP was considered a potential confounder in our current analyses. On this basis, we excluded patients with IHP from our analyses.

In our study, NIHSS score on admission was still associated with poor short‐term outcome in patients with mild AIS. NIHSSs can sensitively stratify the severity of neurological deficits in patients with mild anterior circulation strokes. In addition, we did not exclude patients with posterior circulation strokes and NIHSSs may not be sensitive enough to evaluate the neurological deficits in patients with posterior circulation strokes. For those patients, small NIHSS differences may actually indicate neurological deficits which affect the clinical outcome.

In the present study, we identified ISILAO as a predictive factor of the short‐term clinical outcome, a finding in line with that in a previous study. (Nedeltchev et al., 2007).

The current study has several strengths. First, to our knowledge, it is one of only a few studies that have focused on the association between high NLR and short‐term poor outcome in patients with mild AIS receiving IVT. Second, we evaluated patients at discharge and 3 months after the onset of stroke. Therefore, our analysis provides a comprehensive method for evaluating the predictive potential of NLR with regards to poor short‐term outcomes. Third, IHP, a potential confounder, was excluded from our analysis, thus ensuring that our findings were as reliable as possible. Finally, an advantage of our study over many previous studies is that we included neuroimaging variables in our analysis. However, our study also has some limitations that need to be considered. First, the sample size of this study was relatively small. Second, we lacked dynamic NLR data; the inclusion of such data may enable more reliable predictions. Third, we only excluded patients with infection before admission via self‐reporting and symptoms.

## 5 CONCLUSION

Our analyses demonstrate that a high NLR serves as a useful predictor of poor short‐term outcome in patients with mild AIS. Further prospective studies with larger sample sizes and dynamic NLR data are now warranted.

## CONFLICT OF INTERESTS

None declared.

## AUTHORS' CONTRIBUTIONS

Yong‐Lin Liu and Yang‐Kun Chen participated in the conception and design of the study, the analysis of clinical data, and critical revision of the manuscript for scientific validity. Zhi‐Qiang Wu, Jian‐Feng Qu, Dong‐Hai Qiu, and Gen‐Pei Luo, helped to acquire raw data. Han‐Peng Yin followed up the patients. Xue‐Wen Fang and Fang Wang analyzed the imaging data. All authors have read and approved the final manuscript.

### Peer Review

The peer review history for this article is available at https://publons.com/publon/10.1002/brb3.1857.

## Data Availability

The data that support the findings of this study are available from the corresponding author upon reasonable request.
